# Multi-feature fusion based face forgery detection with local and global characteristics

**DOI:** 10.1371/journal.pone.0311720

**Published:** 2024-10-10

**Authors:** Yuanqing Ding, Fanliang Bu, Hanming Zhai, Zhiwen Hou, Yifan Wang

**Affiliations:** 1 School of Information Network Security, People’s Public Security University of China, Beijing, China; 2 Department of Criminal Investigation, Sichuan Police College, Luzhou, China; Tianjin University, CHINA

## Abstract

The malicious use of deepfake videos seriously affects information security and brings great harm to society. Currently, deepfake videos are mainly generated based on deep learning methods, which are difficult to be recognized by the naked eye, therefore, it is of great significance to study accurate and efficient deepfake video detection techniques. Most of the existing detection methods focus on analyzing the discriminative information in a specific feature domain for classification from a local or global perspective. Such detection methods based on a single type feature have certain limitations in practical applications. In this paper, we propose a deepfake detection method with the ability to comprehensively analyze the forgery face features, which integrates features in the space domain, noise domain, and frequency domain, and uses the Inception Transformer to learn the mix of global and local information dynamically. We evaluate the proposed method on the DFDC, Celeb-DF, and FaceForensic++ benchmark datasets. Extensive experiments verify the effectiveness and good generalization of the proposed method. Compared with the optimal model, the proposed method with a small number of parameters does not use pre-training, distillation, or assembly, but still achieves competitive performance. The ablation experiments evaluate the role of each component.

## 1 Introduction

Deepfake is a ’fake’ image or video generated by ’deep’ learning algorithms. Due to the simple production, low cost, and rapid dissemination of deepfake videos, the number and attention of deepfake videos has increased rapidly, and it is reported that in global mainstream websites and social media platforms, the number of newly released deepfake videos in 2021 has increased by more than 10 times compared with that of 2017 [1]. With the rapid development of deepfake technologies, which provide means for those who maliciously spread false information, deepfake images and videos are becoming more and more realistic, making it difficult for the human eye to distinguish between the real and the fake. Deepfake which blurs the boundary between real and fake will have a huge impact on social trust, media trust, and political trust [2]. Therefore, deepfake detection is of great practical significance.

Detection of deepfake videos is usually regarded as a binary classification problem of video frames. Generally, frames are extracted from the video to be detected, and features in the frame images are analyzed to classify the images as true or false, and thus infer the authenticity of the video. The extraction of features in traditional methods relies on manual design, but it is not effective for videos generated by deep forgeries, therefore, the most current detection adopts deep learning methods, i.e., designing the network architecture and training the network to identify forged features and discriminate the authenticity of videos through supervised learning. The CNN-based network architecture can automatically extract the local features of the image, and use the convolution kernel to process the corresponding region image to get the image features within that receptive field. This works well to detect local inconsistencies existing in the forged image, such as artifacts introduced by tampering operations, and inconsistencies in the edges of tampered and non-tampered regions.

We find that there are still some non-negligible problems with deepfake videos detection in practical applications. First, the forgery detection algorithms fail to keep up with trends. With the continuous progress of forgery technology, the quality and fidelity of the forged content are constantly improving, which makes the existing forgery detection algorithms invalid. For example, in face forgery videos generated by GAN networks, artifacts and jitters in local regions are gradually reduced, so detection methods relying only on mining local regions for anomalies are no longer effective. More forgery cues feature inconsistencies between local regions, like the lighting of the synthesized eye and mouth regions may not be consistent with the lighting of the face region. To address this problem, some studies adopted the Visual Transformer model (ViT) [3] to develop a global perspective to analyze the features of deep forgery images by constructing the relationships of distant image blocks. However, the detection performance is not satisfactory when simply porting the ViT to the deepfake detection task. Not to mention the large model size and complex computational structure of the Transformer, it requires more computational resources and storage space, which will limit its application in some realistic scenarios. Second, most of the existing forgery detection algorithms are poorly generalized. The performance of the algorithms drops dramatically in the face of unknown counterfeiting means in practical applications. Since different forgeries tend to leave specific traces or anomaly patterns in different domains, for example, some forgeries may not be obvious in the RGB domain but may show significant discriminative properties in the frequency domain or other domains. Therefore, extracting a single form of image features is not enough to cope with the ever-changing forgery methods, which is an important reason for the relatively poor generalization performance of the current algorithms.

To solve these problems, we propose a simple but effective network architecture that combines the advantages of CNN and Transformer networks. It extracts the global and local information of the video to discriminate the authenticity of the video. Specifically, the video is first converted to frames and the face images are cropped, and then the images are fed into the Multi-Feature Extraction module to obtain the spatial domain features, noise features, and frequency domain features of the face, respectively. A fusion of these three features is performed and then fed into the Inception Transformer module [4] to accomplish the true and false classification. Extensive experiments on commonly used deepfake detection datasets confirm the effectiveness of the proposed method within and across datasets. The method achieves competitive results compared to existing baseline models. Notably, we do not use any pre-trained models or techniques such as distillation learning, and the number of parameters in our model is much lower than that of existing state-of-the-art models.

Our contributions in this paper are as follows. 1) Applying the Inception Transformer model to deepfake videos detection and verifying its effectiveness on several deepfake detection benchmark datasets. It suggests that the joint use of global and local information is a good idea for the detection of forged videos. 2) A Multi-Feature Extraction (MFE) module is designed, which fuses texture features, noise features, and frequency features, while suppresses semantic information to make the model more sensitive to forgery traces. Experiments show that the MFE module is beneficial in improving the deepfake detection performance. 3) Proposed a lightweight model, named Mixformer, that consists of the underlying MFE module and Inception Transformer for forgery face detection. The model has a much smaller number of parameters, less than one-fifth of the state-of-the-art model. And the model’s performance remains competitive without the use of pre-training, distillation, or assembly. This indicates that our model is more efficient and practical.

The rest of the paper is structured as follows: Section 2 describes related work in the area of deepfake generation and detection. The proposed Mixformer model is described in detail in Section 3. The experimental procedures and results are described in Section 4. Finally, conclusions are drawn in summary in Section 5.

## 2 Related work

### 2.1 Deepfake faces generation

Deepfake originally originated from the user ’deepfakes’ who posted fake videos on the Reddit social network in 2017. With the development of deep learning technology, there are currently three main ways to generate deepfake faces: based on Variational Auto Encoders (VAE), based on Generative adversarial networks (GAN), and based on Diffusion Models (DM) approaches.

The autoencoder consists of an encoder and a decoder, the generated face retains the identity of the source face and also has the expression features of the target face. However, the reality of the face generated by the autoencoder network is limited. Deepfake face generation based on GAN networks is the result of the mutual fight between generators and discriminators. ProGAN [5] proposes to train the generator and discriminator step by step by progressively deepening the network for face synthesis. The StyleGAN family [6,7] redesigns the generator architecture to control the style of the face synthesis results. FSGAN [8] can exchange faces in the video in real-time. FaceShifter [9] extracts the attributes of a multilevel target face by attribute encoding, uses the generator to adaptively embed identity categories and attributes for face swapping, and restores regions with occlusions through a self-supervised approach to generate high-fidelity faces. Diffusion model [10] first gradually adds noise to the data through a forward process, then predicts the noise added at each step through a reverse process, and removes the noise to gradually restore the noise-free image. Common models include DALL-E2 [11], Stable Diffusion [12], etc. In practice, the face-swapping function of Roop software has added the Stable Diffusion plug-in.

### 2.2 Deepfake detection

The increasing number of high-quality deepfake videos brings risks to social security. To cope with it, many deepfake detection methods have been proposed. D. Afchar et al. [13] proposed MesoNet to automatically and efficiently detect fake faces in videos using meso-level image features. A facial X-ray based face image forgery detection [14] is proposed using the hybrid boundary introduced by the forged face embedded in the real image during the tampering process. The detection algorithm remains effective when the tampering technique is unknown. Zhao et al. [15] regard this task as a fine-grained classification problem and propose a multiple spatial attention mechanism to aggregate the low-level textural feature and high-level semantic features. Liu et al. [16] proposed a residual Federated learning to learn robust discriminative residual feature maps to detect forgery faces. Wodajo et al. [17] applied the convolutional Visual Transformer (ViT) to deepfake videos detection, a CNN network is used to extract learnable features, which are then fed into a ViT network, which employs an attention mechanism to classify the feature. Since the ViT network deals with image features rather than images, this drastically reduces the dimensionality of the network and improves the training speed of the network. The method was trained on the DFDC dataset and achieved competitive results, but it did not generalize well over the FaceForensics++ FaceShifter dataset. A two-branch method [18] is proposed, where images are fed into two EfficientNet B0 feature extractors separately, and the obtained image features are then fed into visual transformer encoders at different scales, and the outputs of the encoders are fused using cross-attention. The method is trained on DFDC and FaceForesics++ datasets and tested on the DFDC dataset, it achieved state-of-the-art AUC values, but the architecture has a large number of parameters, a large amount of training data, and a high computational cost.

After the propagation of different streams on the network, videos and images are usually compressed several times, which makes the forgery artifacts more difficult to recognize. Therefore, unlike the detection methods based on the spatial domain [13–18], researchers try to obtain the detection clues from other perspectives such as the frequency domain. For example, Qian et al. [19] found that the details of artifacts brought by the forgery method can be well mined in the frequency domain, and the method still maintains excellent detection performance in the face of highly compressed forgery images. Wang et al. [20] explore the spatial-temporal characteristics in the frequency domain for VIS and NIR scenario. Furthermore, to fully exploit the rich information in video sequences or images, detection methods utilizing multiple features are proposed. Liu et al. [21] combine spatial image and phase spectrum to capture the up-sampling artifacts of face forgery to improve the transferability. Peng et al. [22] use gaze features in conjunction with texture and attribute features of video sequences to enhance the representation of spatial-temporal feature differences between real and forged faces.

## 3 Methods

### 3.1 Overview

In this section, we first state the motivation for designing our method and then provide a brief overview of it. As aforementioned, ViT networks have achieved good performances in deepfake detection tasks. ViT-based deepfake detection methods utilize long-distance relations among image patches to capture global information and discover forgery clues. In fact, the traces of forgery may be global or local. Over-propagation of global information will strengthen the low-frequency representation, deteriorate the high-frequency part such as local texture, and weaken the modeling ability of ViT [23]. To solve this problem, the forgery detection network should learn both the global and local information of the image. The Inception Transformer proposed in the literature [4] fulfills this requirement exactly. It can effectively learn comprehensive features both globally and locally by incorporating the advantages of CNNs in capturing high-frequency information into ViT. Therefore, the Inception Transformer is chosen as the backbone network for the deepfake detection task in the method.

Meanwhile we observe that forgery traces are subtle and vary from case to case. Utilizing multiple types of features to analyze forgery traces is a comprehensive approach. For example, Zhou et al. [24] propose a two-stream network that utilizes both RGB stream and noise stream for image tampering detection. Lin et al. [25] propose a novel network to learn and enhance multiple tampering traces, including noise distribution and RGB visual artifacts. Liu et al. [21] combine spatial image and phase spectrum.

**Fig 1:**
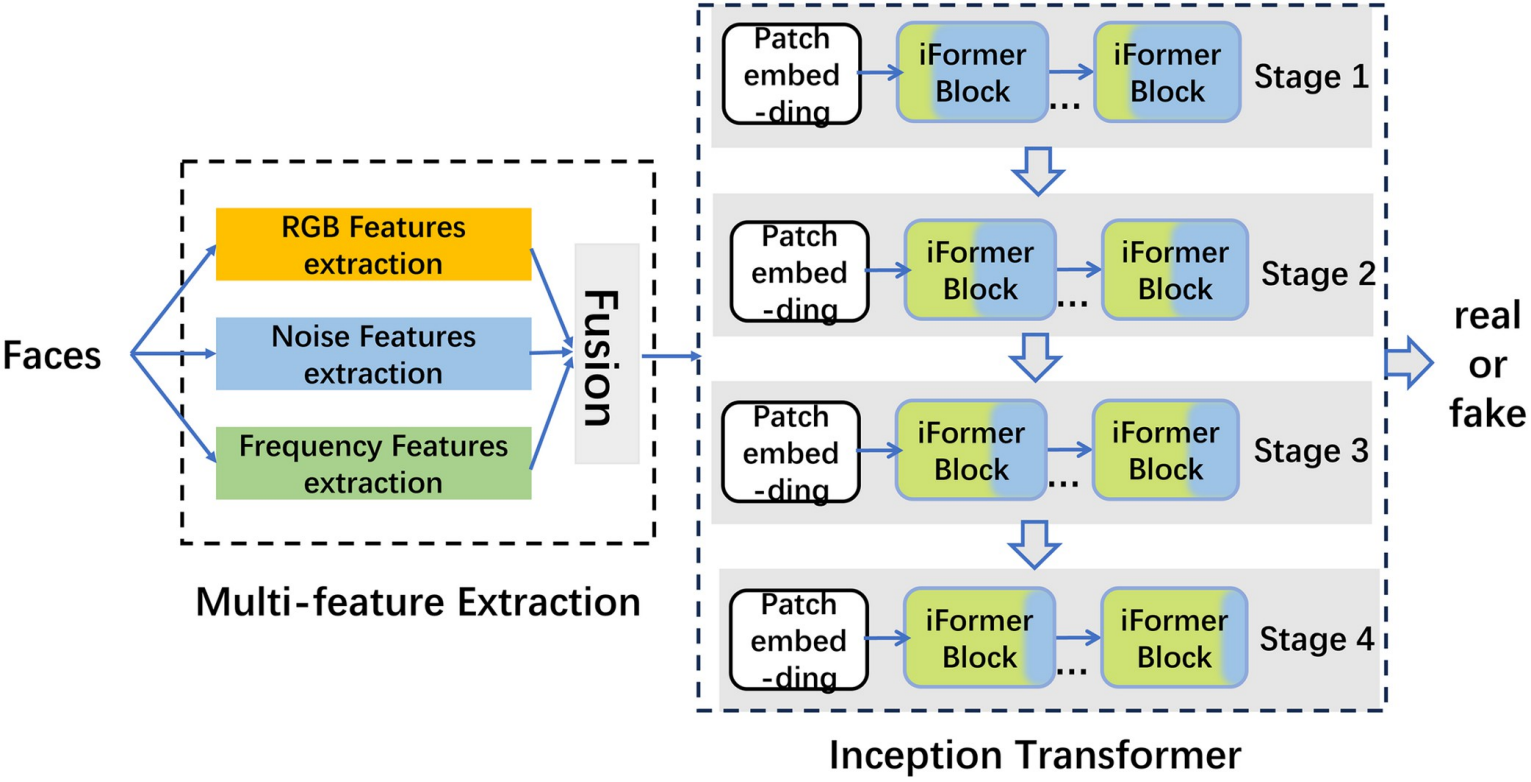
The overall structure of Mixformer model.

Motivated by these observations, we propose a novel deepfake detection model, named Mixformer, to exploiting forged features of image faces in different representation domains. The proposed Mixformer consists of two key components: 1) a Multi-Feature Extraction (MFE) module, integrates features in the space domain, noise domain, and frequency domain, enable the model learn better forgery traces. 2) an Inception Transformer module, learns the mix of global and local information dynamically. The overall architecture diagram is shown in Fig 1. The detailed steps for each of these two sections are described separately below.

### 3.2 Multi-feature extraction module

Convolutional networks are used to extract image semantic features, while the basis of image identification is subtle and often unrelated to semantics, such as mixed boundaries, inconsistency between the forged region and the real region, etc. Therefore, for the task of image forgeries, it is not enough to feed the image into a simple convolutional neural network to extract features for analysis. In this paper, a hybrid feature extraction module is designed to enhance the network’s ability to identify forged traces by integrating the conventional features, noise features, and frequency domain features of the image.

**Fig 2:**
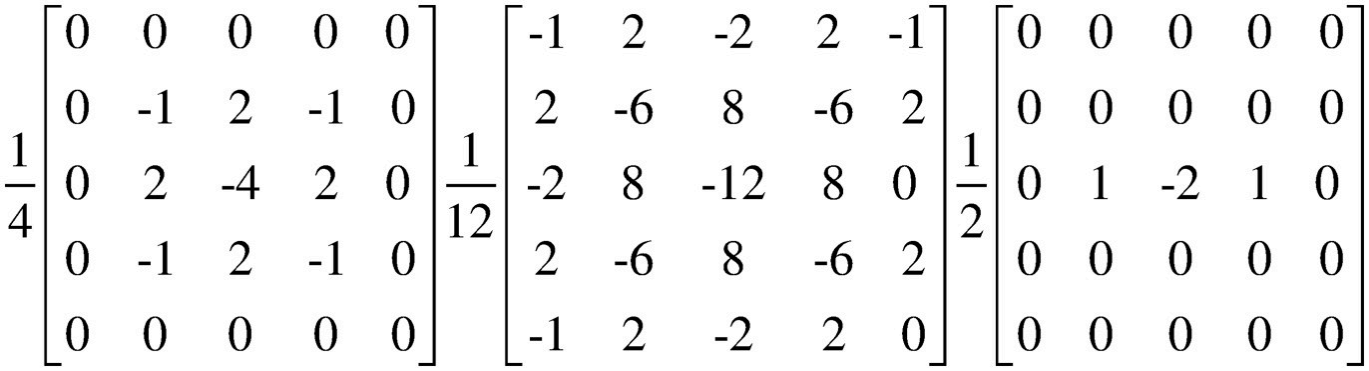
SRM filter kernels for extracting noise features.

Firstly, as images from the same source or produced by the same device have the same noise pattern, the noise feature can be considered as an inherent specificity of the image. The forgery operation destroys the consistency of features in the original image and thus usually leaves special traces in the noise space [26,27]. Inspired by this, in this paper, we use the Steganalysis Rich Model (SRM) to extract the high-frequency noise of an image and use it as one of the discriminative features for forgery traces. We use a fixed SRM filter [24,28] with 3 SRM cores as shown in Fig 2. Three channels of RGB image are passed through the SRM layer with 3 cores respectively to obtain the noise features that reflect the incongruence between the real and the tampered regions.

The fixed SRM filters have limitations because of manually designed. Therefore, to adaptively learn features from the image noise space and further suppress the influence of the image content on the tampering traces, we added the constrained convolutional layer BayerConv2D [29]. The kernel size of Bayerconv2D is 5 and the number of filters is 3.

Secondly, tampering operations may change the frequency domain features of an image. Qian et al. [19] adopts frequency aware decomposition (FAD) to separate the high-frequency, mid-frequency, and low-frequency parts of an image. Inspired by this, we allow the network to learn the different features of the forged image and the real image in these three parts, thus further enhancing the discriminability of the features. Specifically, the image is first transformed to the frequency domain by DCT transformation. Then it is segmented into low-frequency part, mid-frequency part, and high-frequency part using a filter. These three parts are then cascaded after being transformed to the RGB domain by IDCT transform, respectively. Following the original paper [19], the low-frequency sub-band is 1/16 of the entire spectrum, the mid-frequency sub-band is 1/16 to 1/8, and the high-frequency sub-band is the remaining 7/8.

This work starts by extracting frames from the video, converting the video into a sequence of frames, and then cropping these frames to keep only the face region to get the face images. Assuming that a face image is represented as X, X is the input to the hybrid feature extraction module and is fed into the classical Conv2D layer, SRMConv2D, BayerConv2D [29], and the FAD module [19], respectively, to obtain a variety of features. These features are then cascaded to obtain hybrid features. This process is expressed as Eq (1):

$$X_{mix}=Concat\left(Conv\left(X\right),SRM\left(X\right),BayarConv\left(X\right),FAD\left(X\right)\right)$$ (1)

Where

*X* ∈ *R*^3,*H*,*W*^ is an input image tensor with 3 channels (RGB) and dimension *H* (height) and *W* (width).

*X*_*mix*_ ∈ *R*^*C*,*H*,*W*^ is the output hybrid feature.

*C* represents the total number of channels of in *X*_*mix*_, derived from the concatenation of features from Conv, SRM, BayarConv and FAD operations.

**Fig 3:**
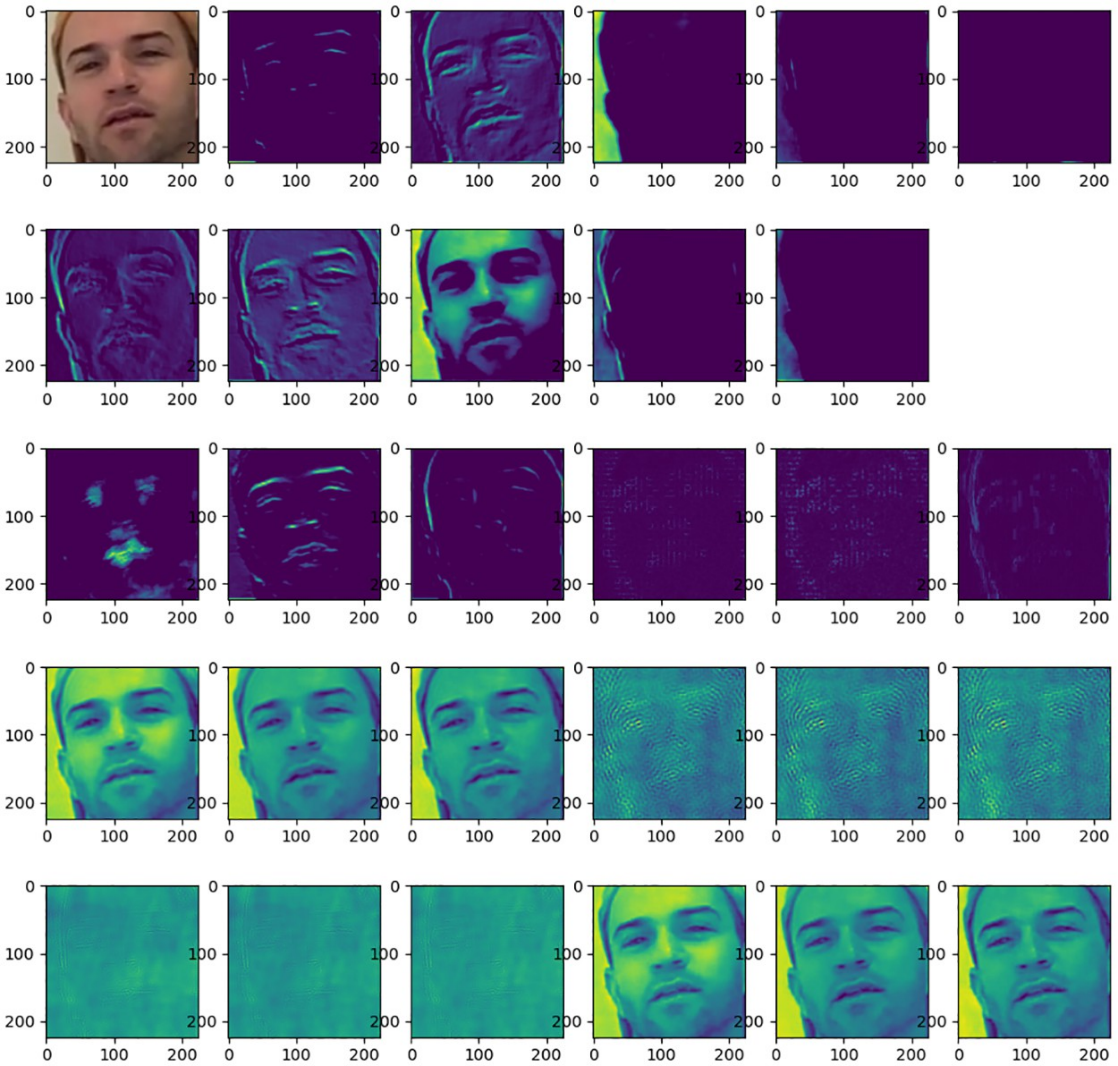
Various feature maps of a real face. The top left corner displays the RGB image of a frame face from the public Deepfake Detection dataset, the rest of the first two rows are spatial domain features, the third row is the noise domain feature map, and the fourth and fifth rows are the frequency domain feature maps.

**Fig 4:**
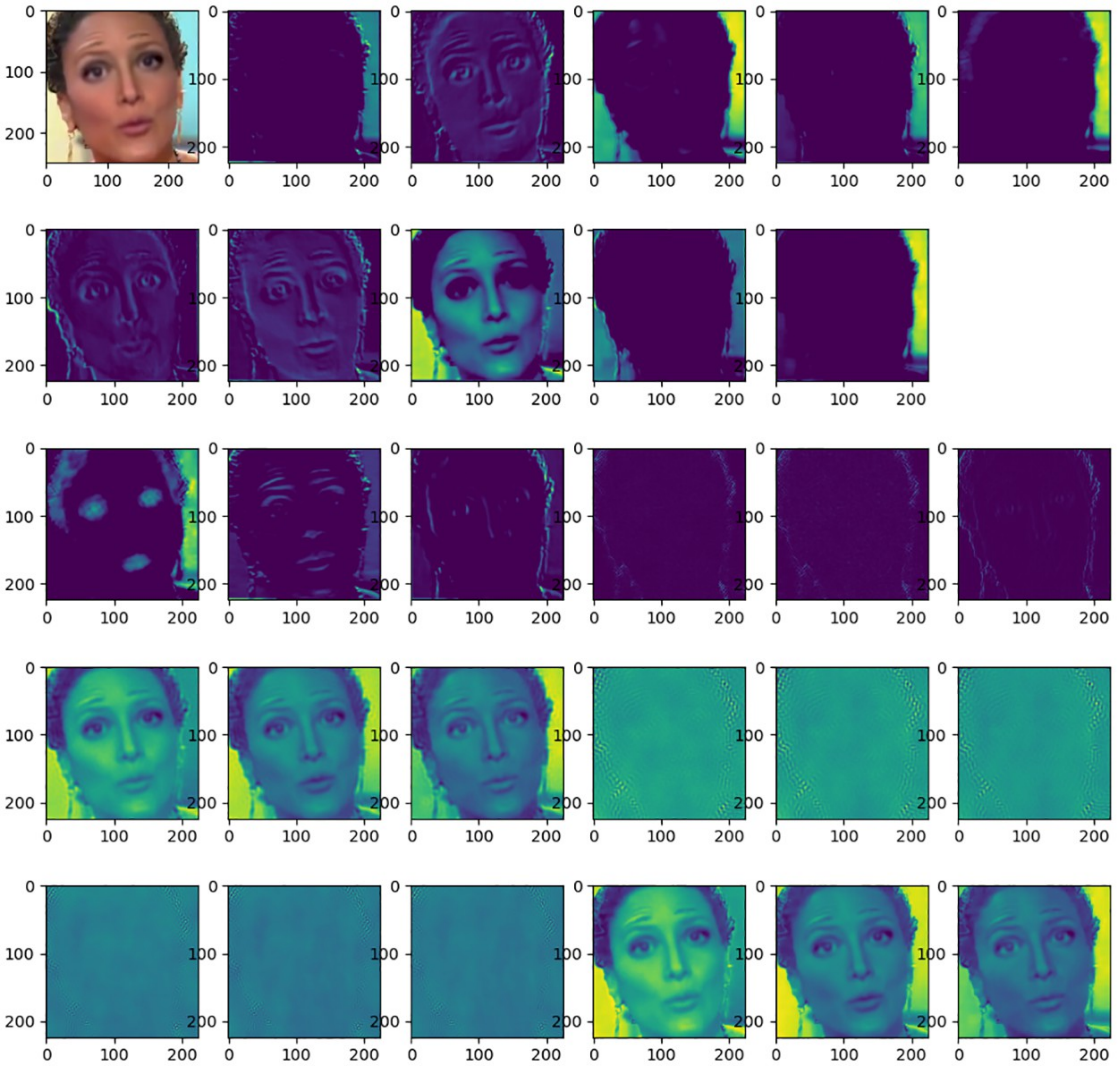
Various feature maps of a fake face. The top left corner displays the RGB image of a frame face from the public Deepfake Detection dataset, the rest of the first two rows are spatial domain features, the third row is the noise domain feature map, and the fourth and fifth rows are the frequency domain feature maps.

The hybrid feature maps corresponding to real face and forged face images are given as shown in Figs 3 and 4 respectively. The real face and the forgery face are sourced from the public Deepfake Detection Challenge dataset. Where the top left shows the RGB image, the subsequent first and second rows show the convolutional features, the third row shows the noise features of the image including Bayer features and SRM features, and the last two rows show the frequency domain features of the image. As can be seen from the figures, the convolutional features mainly extract the content information of the image and have a good representation of the semantic information of the face. While Bayer features and SRM features mainly extract noise features of the face. The middle and high-frequency domains of the frequency domain features reflect the details and edge information of the image. Comparing the feature maps of the real face and fake face, it can be found that the blending boundary of the fake face is more obvious in the noise domain, and there is a significant difference between the face texture of the real face and the fake face in the middle and high-frequency domains, which is very difficult to find in the RGB domain. From this, we can see the necessity of this multi-feature extraction module.

### 3.3 Inception transformer module

Literature [4] suggests that a network for understanding images should capture more high-frequency detail information at lower layers, and gradually increase the low-frequency global information as the number of network layers increases, just like human beings achieve a global understanding of images by gradually collecting local information. This idea coincides with the task of image forensics, which gradually expands from observing the subtle points of the image to the global information to comprehensively judge the authenticity of the image. Inspired by this, this paper adopts the Inception Transformer network proposed in that literature and uses the multi-feature generated above as inputs to learn the forgery traces from local to global, and outputs binary classification values.

**Fig 5:**
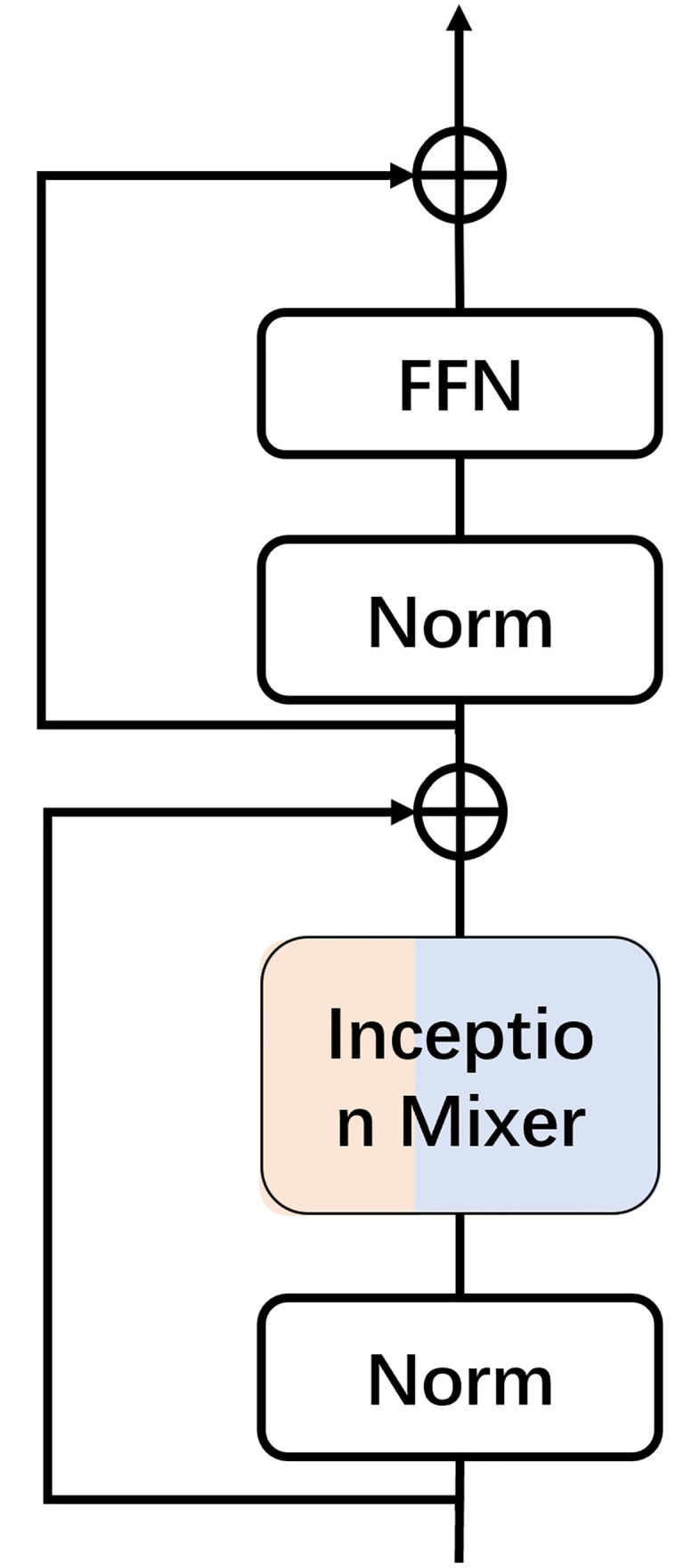
iFormer block structure diagram.

The Inception Transformer module consists of four stages, each of which consists of a patch embedding and iFormer blocks as shown in Fig 1. The specific structure of the iFormer block is shown in Fig 5. The iFormer also has a feed-forward network (FFN) like a common Transformer, with the difference of incorporating the Inception Token Mixer (ITM). Layer normalization (LN) is used before ITM and FFN. For each block, the channel rate determines the allocation of the HF and LF portions, i.e., Ch/C and Cl/C, where Ch/C + Cl/C = 1. This structure gradually splits more channel sizes from lower to higher layers to the LF mixer, thus reducing the channel size of the HF mixer. As can be seen from the color of the iFormer in the figure, Ch/C gradually decreases in the blue part and Cl/C gradually increases in the yellow part from the lighter to the deeper layers. In this way, the iFormer effectively balances the high and low-frequency parts between all layers.

**Fig 6:**
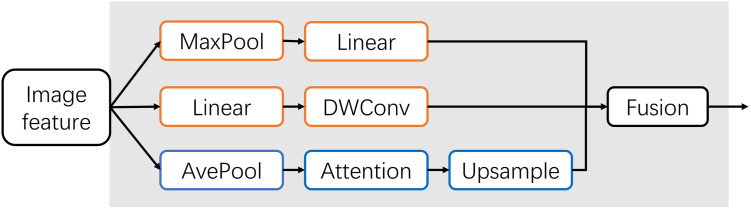
Inception token mixture structure diagram. Where the orange box indicates the high-frequency mixer part and the blue box indicates the low-frequency mixer part.

The important component of iFormer is the inception Token mixer, as Fig 6 Shown. This mixer segments the input features along the channel dimension and feeds the segmented parts into the high-frequency mixer and low-frequency mixer respectively. The high-frequency mixer is responsible for extracting local information through pooling and convolution operations, and the low-frequency mixer is implemented by self-attention in the ordinary ViT, which is responsible for learning the relationship of global information. In this way, the network can efficiently capture frequency-specific information on the corresponding channel. It can learn features over a wider frequency range than with a regular ViT.

To better understand the iFormer block, we can represent it by Eqs (2) and (3):

$$Y=X_{mix}+ITM\left(LN\left(X_{mix}\right)\right)$$ (2)

$$H=Y+FFN\left(LN\left(Y\right)\right)$$ (3)

Where *ITM*(∙) denotes the inception mixer. The mixing features *X*_*mix*_ are first linearly projected to obtain the feature mapping *X*_*proj*_ ∈ *R*^*N*×*C*^. *X*_*proj*_ is partitioned along the channel dimension into $X_{h}\in R^{N\times C_{h}}$ and $X_{l}\in R^{N\times C_{l}}$, where *C*_*h*_ + *C*_*l*_ = *C*, and then *X*_*h*_ and *X*_*l*_ are assigned to the high-frequency mixer and low-frequency mixer, respectively.

In the high-frequency mixer, the input *X*_*h*_ is split in two along the channel, i.e., $X_{h1},X_{h2}\in R^{N\times C_{h}/2}$. The two parts are fed into two parallel branches, *X*_*h*1_ through a maximal pooling and a linear layer, and *X*_*h*2_ is fed into a linear layer and a depthwise separable convolution layer. In this way, the high-frequency mixer outputs are *Y*_*h*1_ and *Y*_*h*2_, as shown in the following equations.


$$Y_{h1}=\text{FC}\left(MaxPool\left(X_{h1}\right)\right)$$ (4)

$$Y_{h2}=\text{DwConv}\left(FC\left(X_{h2}\right)\right)$$ (5)

The low-frequency mixer first performs average pooling to reduce the spatial dimension of *X*_*l*_, then employs a common multi-head self-attention operation, and finally performs up-sampling to restore the original spatial dimension. This design drastically reduces the computational overhead and allows the attention operation to focus on global information. The output of the low-frequency mixer can be represented as:

$$Y_{l}=\text{Upsample}\left(MSA\left(AvePooling\left(X_{l}\right)\right)\right)$$ (6)

Next, the outputs of the low-frequency and high-frequency mixers are cascaded along the channel dimension to obtain *Y*_*c*_, followed by depth-separable convolution and a cross-channel linear layer to obtain the output *Y* of the inception mixer.


$$Y_{c}=Concat\left(Y_{l},Y_{h1},Y_{h2}\right)$$ (7)

$$Y=FC\left(Y_{c}+DwConv\left(Y_{c}\right)\right)$$ (8)

Then it goes through a layer normalization (LN) and feed-forward network (FFN). Like the vanilla Transformer encoder, we also use a residual connection, as in Fig 5. Hence the Inception Transformer block is formally defined as:

$$\text{H}=\text{Y}+\text{FFN}\left(LN\left(Y\right)\right)$$ (9)

The proposed model Mixformer consists of four main stages, each of which consists of a stack of the Inception Transformer blocks. After four stages, the finally CLS tokens are used to generate binary classification values.

## 4 Experiments

In this section, we use 3 public available datasets to evaluate the performance of proposed method on deepfake detection task. All experiments run on a computer with NVIDIA Geforce RTX 3080 10GB GPU. We use Pytorch to implement our methods.

### 4.1 Experimental parameters setting

Table 1 shows the detailed configuration of the proposed model. The model uses a 4-stage architecture. Ch/C and Cl/C in the table denote the percentage of the high-frequency portion and the percentage of the low-frequency portion, respectively, and from shallow to deep, Ch/C decreases gradually while Cl/C increases gradually. The kernel size of the Depthwise separable convolution kernel and maximum pooling is set to 3 × 3 in the high-frequency mixer. This model is trained using a binary Cross-Entropy Loss function. A small batch of 32 images was normalized with a mean of [0.485,0.456,0.406] and a standard deviation of [0.229,0.224,0.225]. The normalized face images were then augmented before each training of the model. An Adam optimizer with a learning rate of 0.1e-3 and weight decay of 0.1e-6 was used for optimization. The model was trained for a total of 50 epochs. The learning rate was reduced by a factor of 0.1 at a per-step length of 15.

**Table 1:** Parameters configuration of the iFormer blocks. Pool stride denotes the stride of the pooling and upsample layers in the attention branch.

Layer	Stage 1	Stage 2	Stage 3	Stage 4
Patch embedding	3×3, stride 2,48 3×3, stride 2,96	2×2, stride 2,192	2×2, stride 2,320	2×2, stride 2,384
iFormer block	$\left[\begin{array}{l} \frac{C_{\text{h}}}{C}=\frac{2}{3}\\ \frac{C_{\text{l}}}{C}=\frac{1}{3}\\ pool\;stride\;2 \end{array}\right]\times 3$	$\left[\begin{array}{l} \frac{C_{\text{h}}}{C}=\frac{1}{2}\\ \frac{C_{\text{l}}}{C}=\frac{1}{2}\\ pool\;stride\;2 \end{array}\right]\times 3$	$\left[\begin{array}{l} \frac{C_{\text{h}}}{C}=\frac{3}{10}\to \frac{1}{10}\\ \frac{C_{\text{l}}}{C}=\frac{7}{10}\to \frac{9}{10}\\ pool\;stride\;2 \end{array}\right]\times 3$	$\left[\begin{array}{l} \frac{C_{\text{h}}}{C}=\frac{1}{12}\\ \frac{C_{\text{l}}}{C}=\frac{11}{12}\\ pool\;stride\;2 \end{array}\right]\times 3$

### 4.2 Datasets

To make the experimental results fair, we use publicly available benchmark datasets for deepfake detection: Deepfake Detection Challenge (DFDC) [30] and FaceForensics++ (FF++) [31]. The DFDC dataset is a dataset of face-swapped videos released in the deepfake detection challenge hosted by Facebook. It comes with 23564 videos taken by 3426 subjects and then 104,500 fake videos generated by eight deepfake techniques, with a total data volume of up to 472 G. The FF++ dataset contains 1,000 real videos from YouTube and sub-datasets of fake videos generated by utilizing five deepfake techniques respectively: Deepfakes, DeepFakeDetection, Face2Face, FaceSwap, and NeuralTextures. The dataset contains three different quality versions: the original version without any post-processing (denoted as FF++RAW), the compressed video with a fixed rate quantization parameter of 23 (denoted as FF++HQ), and the compressed video with a fixed rate quantization parameter of 40 (denoted as FF++LQ). Thus, for each quality version, there are 1000 real videos and 5000 fake videos. The HQ version is used in this experiment. We also used the Celeb-DF dataset [32], which consists of 590 real videos of celebrities from the web and 5639 faked videos.

Table 2 gives the split size of the relevant datasets in the experiments, i.e., the number of videos used for training, validation, and testing, respectively. As an example, the proposed model is trained using the DFDC dataset, and faces are extracted using the BlazeFace Neural Face Detector [33], MTCNN [34], and Face Recognition DL libraries. The face images are stored in JPEG file format with an image resolution of 224 x 224. A compression ratio of 90% is also applied. The number of face images in the training set, validation set, and test set are 172245, 64592, and 16320 respectively. Each true and false class has almost the same number of images in all sets. We use Albumentations [35] for data enhancement.

**Table 2:** Train, validation and test split size used in this experiment. The count refers to the number of videos.

Dataset	Training set	Validation set	Test set
Deepfakes(FF++)	1,800	100	100
FaceSwap(FF++)	1,800	100	100
Face2Face(FF++)	1,800	100	100
FaceShifer(FF++)	1,800	100	100
NeuralTextures(FF++)	1,800	100	100
Celeb DF	4,571	980	978
DFDC	81,827 (172,245 images)	24,808 (64,592 images)	12,556 (16,320 images)

### 4.3 Evaluation metrics

For each video to be detected, 30 facial images are extracted and passed to our trained model, which determines the authenticity of the video based on the mean probability of authenticity of these images. We evaluate our model using the average of the accuracy ACC, AUC, F1 score, and loss value over the test dataset. AUC [36] is the area covered by the ROC curve, which is a measure of the evaluation of a binary classification model The process of calculating the F1 value is shown in the following equation:

$$F1=2*\left(Precision*Recall\right)/\left(Precision+Recall\right)$$ (10)

$$Precision=\text{TP}/\left(TP+FP\right)$$ (11)

$$Recall=\text{TP}/\left(TP+FN\right)$$ (12)

Where TP (True Positive) denotes the number of forged images predicted to be forged; FP (False Positive) denotes the number of forged images predicted to be real; and FN (False Negative) denotes the number of real images predicted to be forged. AUC and F1 score both provide a reasonable evaluation of the classifier’s accuracy, even in the presence of sample imbalance.

The loss value indicates how far the prediction result of our model is from the actual target value, and we use the logarithmic loss function to calculate the loss, as shown in Eq (13).

$$LogLoss=-\frac{1}{n}\sum_{i=1}^{n}\left[y_{i}log\left({\hat{y}}_{i}\right)+\left(1-y_{i}\right)log\left(1-{\hat{y}}_{i}\right)\right]$$ (13)

Where *n* is the number of videos to be detected, ${\hat{y}}_{i}$ denotes the estimated probability that the video is a forgery, and *y*_*i*_ is the label of the video with a value of 0 for true and 1 for false.

### 4.4 Experimental results and discussion

#### A. Performance on standard datasets

In this section, we compare the performance of the proposed method with state-of-the-art models such as CViT [17], efficientViT [18], etc. The experimental results of the comparison models are obtained from the results reported in the original paper or from the results of runnings using the public code of the paper.

First, we comparatively analyzed the test results of each model on the DFDC dataset, as shown in Table 3. Among them, the IncepFormer model is the model obtained by feeding the face image directly into the inception transformer network training. The model achieves 80.06% accuracy, 87.1% AUC, and 86.52% F1 value, which confirms that the Inception Transformer can distinguish between real and fake videos more accurately. The proposed Mixformer model has an AUC value of 89.0%, an ACC of 86.73%, and an F1 value of 91.51%, which outperforms the performance of most other models. This demonstrates the effectiveness of the proposed model for the deepfake detection. Compared with the SoTA model CrossViT on the DFDC dataset, the F1 score of the Mixformer model is 3.51% higher than it, up to 91.51%, yet the number of parameters is less than 1/5 of it. The proposed model shows excellent performance in this deepfake detection task with high validity and reliability, and the model also has the lightweight feature, which makes it have wider application prospects and practical value in real situations.

**Table 3:** Comparison of model performance on the DFDC dataset.

Model	Performance (in %)			#parameters
	AUC↑	ACC↑	F1-score↑	(million)
CViT [17]	84.3	-	77.0	89
EfficientViT [18]	91.9	83.2	83.8	109
CrossViT [18]	95.1	-	88	101
Meso-4 [13]	82.2	67.51	-	28
XceptionNet-avg [31]	84.3	-	-	22.8
DSLRFN(AWFs) [37]	83.8	72.14	-	48
IncepFormer(ours)	87.1	80.06	86.52	19
Mixformer(ours)	89.0	86.73	91.51	20

To evaluate the learning ability of the proposed models for different datasets, we also compared the detection accuracy of each model on the sub-datasets of the FF++ dataset: Deepfakes, Face2Face, FaceSwap, FaceShifter and NeuralTextures. The results are shown in Table 4. The proposed model Mixformer has a higher detection accuracy than most models. This indicates that the model can learn the forgery methods of the FF++ dataset well, especially in the FaceShifter sub-dataset, which achieves an accuracy of 98.4%. The detection accuracy of FaceSwap and NeuralTextures decreases due to the fact that, unlike the other tampering that manipulates every frame of the target sequence, these two tampering methods only manipulate the source video and the target video with the minimum number of frames required, resulting in a dip in the accuracy of frame-based detection.

**Table 4:** Comparison of model performance on FaceForensics++ dataset. Validation accuracy (in %).

Model	Deepfakes	Face2Face	FaceSwap	FaceShifter	Neural Textures
Meso-4 [13]	87.27	56.20	61.17	-	40.67
XceptionNet-avg [31]	98.85	98.36	98.23	-	94.5
Baek et al. [38]	71.8	68.6	63.1	-	70.7
Nirkin et al. [39]	94.5	80.3	84.5	-	74.0
ADDNet-3d [40]	92.14	83.93	92.50	-	78.21
SPSL [21]	93.48	86.02	92.26	-	76.78
EfficientViT [18]	83.0	-	78.0	76.0	68.0
CrossViT [18]	87.0	-	84.0	80.0	69.0
Mixformer(ours)	96.8	95.26	72.4	98.4	88.4

#### B. Generalization analysis

Generalization ability is an important metric in machine learning, which refers to the performance of a model when confronted with new scenarios or data. Generalization ability is especially important in deep forgery detection. As forgery techniques evolve and change, the detection model must be able to adapt to the changes and maintain stable performance on unseen datasets.

To evaluate the generalization ability of the proposed model, we perform cross-dataset evaluation between different datasets. As shown in Table 5, the Mixformer model which is trained with DFDC and tested on the Deepfakes subset of FF++ achieves an accuracy of 80.2%. This illustrates that the proposed framework considers DFDC has similar falsification traces with Deepfakes. Whereas, most of the cross-dataset accuracies between the subsets of FF++ are around 50%. One is because the amount of training data is too small, which results in the overfitting of the model; the other is because the differences in the falsification methods of these sub-datasets are quite large.

**Table 5:** Cross-dataset test analysis of Mixformer (evaluated by accuracy in %).

Train	DFDC	Deepfakes	FaceSwap	FaceShifter	Face2Face	NT
Test						
Deepfakes	80.2	96.8	51	-	52	78
FaceSwap	48.5	49	72.4	-	50	48
FaceShifter	46.1	53	59	98.4	49	54
Face2Face	43.8	56.04	50.55	-	95.26	55
NT	48.5	54	46	-	50	88.4

To further compare the generalization ability of the proposed model with other state-of-the-art models, we evaluate the model trained on the DFDC or FF++ dataset by testing it on the Celeb-DF dataset. The results are shown in Table 6. It can be seen that the proposed model achieves the optimal test performance with ACC, AUC and F1 values of 76.71%, 84.1% and 83%, respectively. This confirms that the proposed model is able to recognize tampering traces more accurately facing unseen tampering methods and has more stable detection performance. Meanwhile, the generalization performance of Mixformer is improved by 4.49%, 11%, and 14% over the ACC, AUC, and F1 values of IncepFormer, respectively, which indicates that the multi-feature extraction module greatly improves the generalization of the model. This is due to the fact that the multi-feature module synthesizes the image features in multiple domains, making the extracted features more discriminative. In addition, the model is able to learn both global and local information, which enables the model to understand tampering behaviors at different levels and further improves the generalization ability.

**Table 6:** Comparison of generalizability with other methods (in %).

Model	Train	Test	ACC	AUC	F1
HolisticDFD [41]	DFDC	Celeb-DF	-	70.1	-
CViT [17]	DFDC	Celeb-DF	57.67	55.4	70
Cross ViT [18]	DFDC&FF++	Celeb-DF	52.91	50	69
FCAN-DCT+ResNet50 [20]	FF++	Celeb-DF	-	79.95	-
FCAN-DCT+Xception [20]	FF++	Celeb-DF	-	83.46	-
SPSL [21]	FF++	Celeb-DF	-	76.88	-
Zhao et al. [15]	FF++	Celeb-DF	-	67.44	-
IncepFormer(ours)	DFDC	Celeb-DF	72.22	73.1	69
Mixformer(ours)	DFDC	Celeb-DF	**76.71**	**84.1**	**83**

#### C. Ablation experiments

In this section, the paper uses ablation experiments to verify the effectiveness of the multi-feature extraction module and the Inception Transformer module, we quantitatively evaluated Mixformer and its variants: 1) IncepFormer, which is the Mixformer model without the Multi-feature extraction module, 2) IncepFormer+conv2D, 3) IncepFormer + noise domain feature module, and 4) Mixformer. The results are shown in Table 7.

**Table 7:** Ablation expriments. Comparison between different combinations of Mixformer. The results in the table are test with the DFDC dataset (in %).

No.	Conv2d	Noise feature	Frequency feature	ACC	AUC	F1 Score
1	×	×	×	80.06	87.1	86.52
2	√	×	×	79.80	87.3	86.30
3	√	√	×	81.30	86.9	87.53
4	**√**	**√**	**√**	**86.73**	**89.0**	**91.51**

The IncepFormer model has a slight performance degradation with the addition of the conv2D module, which indicates that the tampering traces of the DFDC dataset are very hard to be detected in the spatial domain, and this is a side effect of the necessity of introducing diversified features. By comparing Model 2 and Model 3, it can be seen that after adding the noise domain feature module, the AUC value does not change much, but the ACC and F1 values both increase by about 1%, which indicates that the noise domain feature helps to improve the deepfake detection ability. After adding the frequency domain feature module to Model 3, the ACC, AUC, and F1 values of the model increased significantly by 5.43%, 2.1%, and 3.98% respectively. These consistently improved performances validate that the proposed noise-domain feature module and frequency-domain feature module indeed help in the detection of deepfakes. Noise domain features and frequency domain features are complementary, hence their fusion further enhances the performance of the model.

#### D. Complexity analysis

To better demonstrate the superiorty of the proposed Mixformer, we also provide model parameters (Param.) and floating point operations (FLOPs) in the paper. Recongnizing that FLOPs only measures the theoretical computational complexity, while the actual inference speed in real scenarios can be affected by hardware equipment and optimization algorithms, we also provide the actual inference speed of the detection model for reference.

Table 8 reports the comparison results of Mixformer with exiting detection method in terms of model parameters, FLOPs and inference time. Note that, the inference times in the table are calculated as the average time taken for the models to infer on each video in the Celeb-DF test set. Among them, the number of parameters in the Mixformer model is one-fifth of that in CrossViT, and although its FLOPs are not the lowest, its actual inference time is shortest. This demonstrates that the proposed model is more lightweight and has broader application scenarios.

**Table 8:** Comparison results of models’ complexity.

Methods	Param. (M)	FLOPs (G)	Inference time (s)
CViT [17]	89	6.68	11.4
EfficientViT [18]	109	0.17	11.4
CrossViT [18]	101	0.17	11.5
Mixformer (ours)	20	4.71	9.8

## 5 Conclusions

In this paper, we propose a lightweight Transformer-based model for deep forgery detection. This model uses Inception Transformer as the backbone network because it can analyze both global and local information, which fits the needs of deepfake detection tasks. By adding spatial, noise and frequency domain features of the image, the model can effectively learn traces of forgery, leading to a gradual improvement in detection performance. On the DFDC and FaceForensic++ datasets, our model does not use any pre-training or distillation methods, but still shows competitive results. On the DFDC dataset, it outperforms the SoTA method using the F1 scores as an evaluation metric. On the FF++ dataset, its ACC performance is close to that of SoTA. The cross-dataset evaluation of the proposed method has better generalization performance than SoTA, which proves that the method has stronger generalization ability. More importantly, the proposed architecture is very lightweight with less than 1/5 of the parameters of the SoTA method. Our study shows that the Inception Transformer is able to distinguish deepfake videos from real videos. The use of fusion features in the spatial, noise and frequency domains greatly enhances the ability of deepfakes detection.
